# Concurrent consumption of cocoa flavanols and caffeine does not acutely modulate working memory and attention

**DOI:** 10.1007/s00394-024-03514-8

**Published:** 2024-11-28

**Authors:** Elkan G. Akyürek, Ahmet Altınok, Aytaç Karabay

**Affiliations:** 1 Department of Experimental Psychology, University of Groningen, Grote Kruisstraat 2/1, 9712 TS Groningen, The Netherlands; 2 Department of Psychology, New York University Abu Dhabi, Abu Dhabi, United Arab Emirates

**Keywords:** Cocoa flavanols, Caffeine, Attentional blink, Visual search, Working memory

## Abstract

**Purpose:**

Consumption of cocoa flavanols and caffeine might acutely enhance cognition, particularly in synergy. Due to the use of multifaceted tasks in prior research, it is unclear precisely which cognitive functions are implicated. Here we aimed to assess the acute effects of the (joint) ingestion of cocoa flavanols and caffeine on temporal attention, spatial attention, and working memory.

**Methods:**

In four separate sessions of a randomized, double-blind, placebo-controlled, crossover trial, 48 young adult participants consumed a placebo drink, a cocoa flavanols (415 mg) drink, a caffeine (215 mg) drink, and a drink containing both concurrently. In each session, after ingestion, we tested performance in three cognitive tasks. We tested temporal attention in a dual-target rapid serial visual presentation paradigm, known to elicit the attentional blink, in which the time between the targets was manipulated. We measured spatial attention in a visual search task, where we varied the number of distractors that appeared simultaneously with the target. We tested working memory in a delayed recall task, in which the number of stimuli to be remembered was manipulated.

**Results:**

We obtained the expected performance pattern in each task, but found no evidence for modulation of response accuracy or reaction times by the ingestion of either substance, nor of their combined ingestion, even in the most challenging task conditions.

**Conclusions:**

We conclude that, even when jointly ingested, neither the tested amount of cocoa flavanols nor caffeine have acute effects that are robustly measurable on cognitive tasks that target attention and working memory specifically.

**Supplementary Information:**

The online version contains supplementary material available at 10.1007/s00394-024-03514-8.

## Introduction

Chocolate and coffee enjoy immense popularity world-wide. They are made from cocoa and coffee beans, in which the psycho-active components of flavanols and caffeine exert acute effects on visual perception, attention, working memory, and executive functions.^1^ These cognitive effects occur through different physiological mechanisms. Cocoa flavanols increase nitric oxide (NO) synthesis [6, 7]. NO binds to guanylate cyclase, triggering structural changes therein, leading to a higher level of guanosine monophosphate in NO generator cells. Consequently, this process prompts vasodilation, affecting both the blood vessels and the cerebral arteries [8]. NO also functions as a pre- and post-synaptic intercellular messenger, which affects neural signaling pathways particularly at GABAergic and glutamatergic synapses mediated by guanylate cyclase [9–11]. NO thereby strengthens communications between neurons and strengthens synaptic plasticity [12, 13]. It should be noted, however, that NO synthesis may require a source of nitrates (cf. [14]). Caffeine blocks A1 and A2a adenosine receptors in various regions of the brain, because of its structural similarity to adenosine [15]. Adenosine inhibits the release of the neurotransmitters glutamate [16], serotonin [17], and dopamine [18]. By blocking of A1 and A2a receptors, caffeine prevents the inhibitory effect of adenosine, effectively stimulating the release of these neurotransmitters.

Cocoa flavanols improve visual perception by enhancing visual contrast sensitivity and acuity [19–21], though see also [22]. Aspects of attention also improve acutely after consumption of flavanols. Among these are higher accuracy and lower reaction time (RT) in the rapid visual information processing (RVIP) and Bakan tasks [23, 24], and lower RT in visual search [25]. Improvements in working memory have been found in serial subtraction tasks [24, 26], in spatial and auditory memory tasks [19, 27], and in N-back tasks [28], although there are several studies reporting null results on spatial and numerical working memory, face recognition, word recognition, and delayed recall [29, 26, 31,30 ]. Lastly, cocoa flavanols have also been reported to improve executive function, as measured in the Stroop task [32], although not consistently so [26, 30, 33]. Task switching performance in particular does not seem to benefit acutely from ingesting flavanols [34].

Caffeine increases color sensitivity during dark adaptation, reduces luminance thresholds [35, 36], reduces surround suppression of perceived contrast [37], and improves dynamic visual acuity [38]. Acute caffeine-induced improvements in attention are found in simple and choice RT tasks [39–41, 42], RVIP tasks [41, 43, 44, 45,2 ], and visual search tasks [46], but not in cueing tasks [47]. It must be noted that some of these positive caffeine effects may be attributed to a reversal of the negative consequences of caffeine withdrawal [48]. Improvements in spatial, verbal, and numeric working memory tasks have been reported [41, 44], [2, 45 ], but not consistently so [39]. Caffeine can even have negative effects on digit span [49], while N-back is only occasionally improved [40, 50–52]. With regard to executive functions, caffeine has been found to reduce task switching costs [53], improve Stroop task performance [54], and reduce RT in the Flanker task [40, 50], although such effects have also been attributed to general speeding [47].

A few studies have been conducted to investigate possible synergistic effects between cocoa flavanols and caffeine. Such synergy is important also from a consumer perspective, as these substances co-occur in commercially available beverages [55]. Synergy was indeed observed by Boolani and colleagues [23], who found that while performance on the Bakan task only improved under dual-task conditions after ingestion of flavanols, single-task performance also improved when combined with caffeine. Another study investigated the concurrent intake of polyphenols from apples and caffeine [56]. Although not identical to cocoa flavanols, apple polyphenols should have similar effects on human cognition. Polyphenols, combined with caffeine, improved serial subtraction performance, beyond a caffeine-related performance improvement, compared to baseline. Although there is thus evidence for acute positive effects of (synergistic) flavanols and caffeine consumption on cognition, the picture is not unequivocal. These mixed results could be due to the tasks used to measure performance. Not only is there variability between tasks, but they also do not always clearly map onto a specific cognitive function. For instance, serial subtraction tasks (e.g., [24] involve working memory to retain and update the numbers, but also involve the ability to do the mathematical transformation, and also require constant attention to keep track of the current number. It remains unclear which of these abilities is eventually affected. Thereby, if another seemingly similar task is used, which does not involve exactly the same abilities (e.g., the N-back task, see also [57]), discrepancies could arise. Here, we avoided this issue by using tasks that are well-defined in experimental psychology to target specific cognitive functions.

## Methods

To assess the cognitive effects of flavanols and caffeine, we used a dual-target rapid serial visual presentation (RSVP) task to measure temporal attention, a visual search task to measure spatial attention, and a delayed recall task to measure working memory maintenance. The canonical RSVP task presents a series of successive stimuli at the center of the screen, at a rate of about 10 per second, and is known to elicit the attentional blink phenomenon at short lag between targets, reflected by poor performance on the second target [58, 59], for a review, see [60], as a consequence of processing the first (e.g., [61–63]). In visual search tasks, the distribution of attention across space, rather than time, is tested. Participants search for a target within an array of simultaneously presented distractors. The time taken to find the target, and to a lesser extent, the accuracy of the search, depends on the ease with which it can be discriminated from the distractors (for an in-depth review, see [64]). Unless the target ‘pops out’ of the array, the number of distractors in the array strongly predicts RT. In delayed recall tasks, working memory is tested, while minimizing attentional factors that often co-occur (e.g., [65, 66]). In these tasks, participants are asked to remember a set of items for a brief time. At the end of the retention interval, the participants are then asked to recall (one of) the items, and response accuracy is assessed (e.g., [67]). The difficulty of this task is manipulated by varying the number of memory items, and performance drops rapidly beyond a set size of four items [68]. In the present study, to assess whether the concurrent consumption of cocoa flavanols and caffeine acutely affects attention across space and time, and/or working memory, we let participants do all three tasks, in four different conditions: Participants either first consumed a drink without psycho-active contents, one with cocoa flavanols, with caffeine, or with both flavanols and caffeine.

### Participants

Forty-eight university students (24 female and 24 male), aged between 19 and 31 ($\overline{X }$ = 21.94, S = 2.50), participated in the study. Further details are given in Table 1. An a priori power analysis in G^*^Power [69] showed that this group size was sufficient to detect an effect of medium size (*f* = 0.30), with two groups and four measurements; α = 0.05; sample size = 24, critical *F* = 2.74 (df = 3). The chosen effect size was based on a previous study by Boolani and colleagues [23], who observed acute effects of cocoa and caffeine on attention, with a ɳ^2^_p_ of 0.085.

**Table 1:** Study sample

Characteristic	N = 48^1^
Gender	Female: 24 (50%), Male: 24 (50%)
Age	21.94 (2.50)
Height (cm)	
Overall	173.67 (10.52)
Female	165.92 (7.68)
Male	181.42 (6.47)
Weight (kg)	
Overall	68.19 (13.90)
Female	59.42 (10.72)
Male	76.96 (10.93)
BMI	
Overall	22.48 (3.48)
Female	21.63 (4.03)
Male	23.33 (2.63)
Handedness	
Right-handed	46 (95.8%)
Left-handed	2 (4.2%)

^1^n (%); Mean (SD)

The participants signed an informed consent form prior to the start of the experiment. The study was approved by the ethical committee of the Psychology Department of the University of Groningen (PSY-1920-S-0472) and conducted in accordance with the Declaration of Helsinki (2008).

The participants met several selection criteria: (1) they were not previously diagnosed with vascular disease, and did not currently have health disorders affecting their metabolism; (2) they had no neurological or psychiatric disorders, and were not following a medically restrictive diet; (3) they did not smoke, and did not use other tobacco products [31, 34], they were not taking over-the-counter or prescription medication, except for the contraceptive pill [24], they were not taking vitamin supplements, herbal extracts, or illicit drugs,(4 they were not currently pregnant or breastfeeding; (5 they had normal or corrected-to-normal visual acuity, and a normal ability to perceive color; (6 they had a body mass index (BMI between 18 and 24.9.

### General apparatus

The data was gathered within the laboratories of the Psychology Department at the University of Groningen. A 22″ CRT monitor with a refresh rate of 100 Hz at a resolution of 1920 by 1200 pixels and 32-bit color depth was utilized during the data collection process. The experimental tasks were created and executed in OpenSesame 3.3.9 [70], running on the Windows 10 system. Responses were collected using a USB keyboard and mouse.

### Experimental product

Participants consumed four experimental ingredients: Caffeine powder, lactose powder, cocoa powder with high flavanol content, and alkalized cocoa powder (see Table 2), which were all dissolved in 200 ml of decaffeinated Nescafe Dolce Gusto capsule coffee (Aromatic Arabica flavor, Lungo serving size, and 6/10 intensity), brewed in a Krups KP1208 coffee machine. In the placebo (P) condition, 7.5 g alkalized cocoa powder and 200 mg lactose powder were administered. In the cocoa flavanols (F) condition, 5 g of high-flavanol cocoa powder (containing 415 mg flavanols), 2.5 g of alkalized cocoa powder, and 200 mg lactose powder were given. In the caffeine (C) condition, 7.5 g of alkalized cocoa powder, and 200 mg of caffeine powder were administered. In the concurrent (CC) condition, 5 g of high-flavanol cocoa powder, 2.5 g of alkalized cocoa powder, and 200 mg of caffeine powder were given.

**Table 2:** Nutritional composition of the study treatments

Consumption conditions					
Experimental ingredients		Placebo	Caffeine	Flavanols	Caffeine + Flavanols
Base Drink: Decaffeinated coffee^1^		one capsule	one capsule	one capsule	one capsule
***1) Alkalized cocoa powder (g)***		***7.5***	***7.5***	***2.5***	***2.5***
Energy (kcal)		22.8	22.8	7.6	7.6
Protein (mg)		1665	1665	555	555
Fat (mg)		825	825	275	275
Caffeine (mg)		15	15	5	5
Theobromine (mg)		157.5	157.5	52.5	52.5
***2) High-flavanol cocoa powder (g)***		***–***	***–***	***5***	***5***
Flavanols (mg)		***–***	***–***	415	415
Energy (kcal)		***–***	***–***	17.2	17.2
Protein (mg)		***–***	***–***	1120	1120
Fat (mg)		***–***	***–***	700	700
Caffeine (mg)		***–***	***–***	10	10
Theobromine (mg)		***–***	***–***	105	105
***3) Caffeine powder (mg)***		***0***	***200***	***0***	***200***
***4) Lactose powder (mg)***		***200***	***0***	***200***	***0***
Energy (kcal)		0.796	***–***	0.796	***–***
Protein (mg)		0.60	***–***	0.60	***–***
Carbohydrates (mg)		190	***–***	190	***–***
Sodium (mg)		0.06	***–***	0.06	***–***
**Total**	**Caffeine (mg)**	**15**	**215**	**15**	**215**
	**Flavanols (mg)**	**0**	**0**	**415**	**415**

^1^Decaffeinated coffee may still contain residual caffeine up to 6.95 mg/8 oz [73]

The bold and italic fonts indicate that these are the main categories (substances), to distinguish them from the other items/subcategories listed

The cocoa powders were provided for free by the Barry Callebaut Company. The company was not otherwise involved in any part of the study. The dosage was based on the study by Karabay and colleagues (2018), in which a similar amount of flavanols had acute effects on visual search efficiency. In terms of the caffeine dose, different strategies have been followed in recent studies. For instance, van den Berg et al. [54] used 3 mg per kg of body weight, while Lanini et al. [71] calculated personalized doses for each participant based on their daily caffeine habits (25–300 mg). In previous research, faster RT was generally observed after a medium to high dose (150–450 mg), while greater accuracy was associated with a low dose of caffeine (50–150 mg [72]. In the present study, we chose a dose of 200 mg of caffeine, which falls in the range that both accuracy and RT may be facilitated, and which is approximately equal to two 16 oz cups of regular coffee [73].

### General procedure

The experiment comprised four sessions with a crossover design so that all participants participated in each consumption condition. The treatment order was randomized with a Latin-square design and gender-balanced (see Supplementary Materials, Table S1). Participants either received a payment of 76 euros, or course credits as compensation. Detailed information about the research, including restrictions in effect prior to, and during the study, was given to participants before their participation. They were furthermore asked not to drink alcohol during a period of 24 h before each of the experimental sessions. To control for circadian effects, the time of day at which participants were tested was kept constant for each individual. Experimental drinks were consumed 90 min before participating in the experimental tasks, to allow for the body’s uptake of flavanol and caffeine [34, 74–76], van den [54]. From the consumption of the experimental drink, until the onset of the first experimental task, the participants were asked not to drink or eat anything except water. To ensure a double-blind procedure, one researcher served the experimental drinks, and another researcher instructed participants in the laboratory.

Participants were seated in individual, isolated testing cabins at approximately 60 cm viewing distance from the computer screen, on which the three experimental tasks were presented to measure temporal attention, spatial attention, and working memory. Task order was also randomized and counter-balanced for each gender separately (see Supplementary Materials). The participants had ample time to read the task instructions, and ask any questions before doing the tasks. In the first session only, the participants filled out a short questionnaire asking about their daily caffeine and flavanols consumption (from cocoa, chocolate and red wine), and their age, gender, body weight, and height. There was a 5–9 days washout period between each session.

### Tasks

**Fig. 1:**
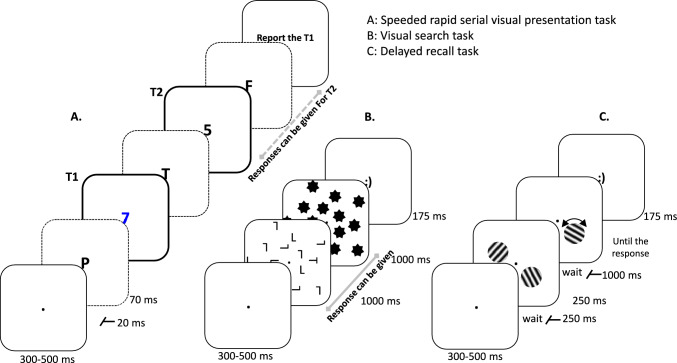
Schematic Overview of the Experimental Tasks. **a** Speeded dual-target RSVP task: Thick-outlined frames highlight the targets (numbers), while dashed outlines signify a varying number of distractors (letters). **b** VS task with 14 distractors. **c** DR task with two memory items

Figure 1 displays the three experimental tasks. Temporal attention was assessed through a speeded rapid serial visual presentation (RSVP) task. Spatial attention was gauged using a visual search (VS) task, while visual working memory was examined by a delayed recall (DR) task.

#### RSVP task

The task consisted of 30 practice trials followed by 300 experimental trials divided into ten blocks. Participants were given breaks between each block. Each trial began with a black fixation dot with an 8-pixel radius (6 pt. size), which was displayed at the center of the screen for 300–500 ms, after participants pressed the spacebar. This was followed by an RSVP stream containing two targets and 16 distractors, shown on a light grey background (RGB 192, 192, 192). Targets were numbers from 1 to 9, while the distractors were 20 uppercase letters (excluding I, O, Q, S, W, and X), both of which were displayed in 52-pt. mono font at the center of the screen. Target 1 (T1) appeared in blue (RGB 0, 0, 255), whereas Target 2 (T2) and the distractors were presented in black. Each stimulus in the RSVP stream was presented for 70 ms, followed by a blank 20 ms inter-stimulus interval. The temporal position of T1 was randomly varied between the 5th and 7th stimulus in the stream, evenly distributed across conditions. T2 followed T1 either as the second (Lag 2), third (Lag 3), or eighth (Lag 8) stimulus. Participants used the numeric keypad of a standard keyboard to report the identity of the targets. They were instructed to identify T2 as quickly as possible, with a 1.5 s time-out. T1 was identified at the end of the trial without time pressure. Feedback on task performance was provided between blocks.

The dependent variables included T1 accuracy, conditional T2 accuracy (T2|T1), and T2 RT for correct T2 responses in conditional T2 trials (T2|T1). Conditional T2 performance refers to trials in which the T1 response was correct. For the T2|T1 RT analysis, responses faster than 100 ms were excluded, resulting in the removal of 290 trials (0.47%).

#### VS task

The VS task included 30 practice trials and 300 experimental trials (100 trials per condition) divided into ten blocks. Each experimental block consisted of 30 trials, and participants were allowed to take breaks between blocks. Each block began when the participant pressed the spacebar. Each trial involved the presentation of a sequence of stimuli on a light grey background (RGB 192, 192, 192), including a fixation dot, search array, mask, and feedback screen. At the beginning of each trial, a fixation dot was displayed for 300–500 ms. The fixation dot was shown in black with a radius of 8 pixels (6 pt. size). It was followed by the search array presented for 1000 ms, and covered with a mask for the next 1000 ms. The search array always contained one target letter (“T”) and a varying number of distractors (“L”; either 14, 20, or 26), displayed in black and 10 pt. size (28 × 60 pixels). The orientation of the letters varied randomly among 0°, 90°, 180°, or 270°, evenly distributed on each trial. All letters were evenly distributed within the search array across three invisible concentric circles. The circles had radii of 100, 150, and 200 pixels (equivalent to 2.53°, 3.79°, and 5.05° of visual angle, respectively) and were centered on the screen. Each invisible circle contained either 5, 7, or 9 letters, placed randomly, but at equidistant locations. In the subsequent masking display, black stars (10 pt. size; 28 × 60 pixels) appeared on locations that had contained letters in the preceding search display.

Participants were instructed to quickly and accurately report the orientation of the target letter. They had a maximum of 2000 ms, until the mask disappeared, to respond with the arrow keys on the keyboard. Following the response, feedback was displayed for 175 ms, represented by either a happy or unhappy smiley, based on their accuracy. The subsequent trial began with an intertrial interval of 250–300 ms after the offset of the feedback display.

Similar to the RSVP analysis, the dependent variables were accuracy and RT for correct responses. As before, RT values below 100 ms were excluded from the RT analysis, resulting in the removal of 81 trials (0.13%).

#### DR task

The task included 30 practice trials and 300 experimental trials, with each condition having 100 trials. Participants had the option to take breaks between blocks if needed. All stimuli were presented on a light grey background (RGB 192, 192, 192). Each trial began with the display of a black fixation dot with a radius of 8 pixels (6 pt. size) at the center of the screen for 300 to 500 ms. After a 250 ms blank interval, the memory array appeared for 250 ms, containing either one, two, or three memory items. Each memory item was shown at 2.75° of visual angle from the fixation dot. The memory items were Gabor patches (sine-wave gratings) with a size of 2.2º of visual angle and a spatial frequency of 1.8 cycles per degree. These memory items were presented on an invisible circle with a diameter of 6.46º of visual angle. The locations of the memory items on the circle were random, following specific constraints. In the two-memory items condition, the items were presented symmetrically on both sides of the visual field. In the three-memory items condition, the items were positioned on an equilateral triangle intersecting the circle. The orientation of each item (ranging from 1 to 180º) was randomly chosen with equal probability in each trial. Following a one-second delay after the memory array, one of the previously shown item locations was probed with the presentation of another grating in a different orientation. This orientation was randomly chosen but at least 15° away from the actual orientation of the target memory item.

Participants were instructed to accurately reproduce the orientation by adjusting the orientation of the probe grating, using a USB mouse. After each trial, response feedback was provided for 175 ms. Positive feedback was given if the error was less than 15°, while negative feedback was given otherwise; either a happy or unhappy smiley in white, 32-pt. size, and mono font type, at the center of the screen. Additionally, participants received block-wise feedback regarding their overall task performance at the end of each block.

The dependent variables were accuracy (%), calculated based on degrees of error, and RT. Trials with an RT of less than 100 ms were excluded from the analysis, resulting in the removal of 30 trials (0.05%).

### Statistical Analysis

Linear Mixed Models (LMM) and Generalized Linear Mixed Models (GLMM) were used to test the acute effects of cocoa flavanols and caffeine on spatial attention, temporal attention and working memory maintenance. Statistical analyses were run in RStudio [77] with the nlme and lme4 [78] packages. The ggplot2 [79] and sjPlot [80] package was used to visualize the results.

In model testing, model improvements comparing simpler models to more complex ones were assessed by testing for differences in deviances (Δd) with a chi-square test. If this test was significant, we then computed the Bayes Factor (*BF*_*10*_; [81]) on the associated Bayesian Information Criterion (BIC) value. We took the following steps: First the initial model (without fixed effects) was tested with random intercepts for subjects. Then fixed effects were added. For the Task conditions these were Lag for the RSVP task (2, 3, or 8), number of Distractors for the VS task (14, 20, or 26), and number of Items (1, 2 or 3) for the DR task. For the Treatment conditions these were placebo, flavanols only, caffeine only, or both flavanols and caffeine concurrently. Additionally, gender and BMI were entered into the final models as fixed effects in a post-hoc analysis stage when main effects or interactions were found. Following the fixed effects analysis, random slopes were added, and pairwise differences were tested with a Tukey test. Practice trials were excluded from analysis in all tasks.

## Results

### Temporal attention

In the GLMM on T1 accuracy, before adding fixed effects, random intercepts for each subject were added to the null model, and the model deviance improved significantly [χ^2^_∆d_ = 1694.6, *df* = 1, *p* < 0.001, *BF*_*10*_ > 100], suggesting that random intercepts for subjects were necessary.

Adding the fixed effect of Lag [χ^2^_∆d_ = 94.19, *df* = 2, *p* < 0.001, *BF*_*10*_ > 100] improved the model significantly. Following that, Treatment was added to the model, and it improved the model significantly [χ^2^_∆d_ = 32.74, *df* = 3, *p* < 0.001, *BF*_*10*_ = 0.827]. However, the interaction of Treatment and Lag did not improve the model [χ^2^_∆d_ = 3.96, *df* = 6, *p* = 0.683, *BF*_*10*_ < 0.001].

**Fig. 2:**
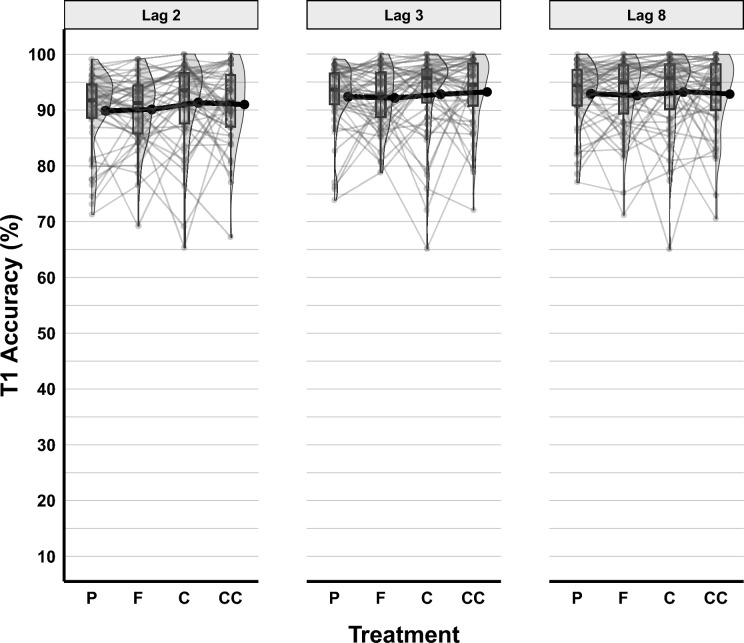
T1 Accuracy in the RSVP Task across Treatment and Lag. Boxplots represent quartiles. Black dots accompanied by error bars depict means and standard errors. Individual data points are illustrated with grey points, and lines connect these individual points across treatments. P refers to the placebo condition, F to cocoa flavanols, C to caffeine, and CC to the concurrent condition (flavanols and caffeine combined)

Random slopes for Lag [χ^2^_∆d_ = 30.72, *df* = 5, *p* < 0.001, *BF*_*10*_ < 0.001] and Treatment [χ^2^_∆d_ = 17.12, *df* = 18, *p* = 0.515, *BF*_*10*_ < 0.001] did not improve the model. Additionally, neither the gender of the participants [χ^2^_∆d_ = 4.62, *df* = 1,* p* = 0.032, *BF*_*10*_ = 0.0040], nor their BMI scores [χ^2^_∆d_ = 0.03, *df* = 1,* p* = 0.853, *BF*_*10*_ = 0.0041], significantly improved the model (see Table S2). T1 accuracy by Lag and Treatment is shown in Fig. 2.

Tukey pairwise comparisons showed that T1 accuracy at Lag 2 (*prob* = 0.928, SE = 0.007, 95% CI [0.912–0.941]) was significantly lower than at Lag 3 (*prob* = 0.945, SE = 0.006, 95% CI [0.932–0.955]), *Z* = 7.87, *p* < 0.001, and at Lag 8 (*prob* = 0.947, SE = 0.006, 95% CI [0.935–0.957]), *Z* = 8.92, *p* < 0.001. There was no significant difference in T1 accuracy between Lag 3 and Lag 8, *Z* = 1.08, *p* = 0.529. Lastly, Tukey pairwise comparisons between Treatment conditions showed no significant differences between them.

**Fig. 3:**
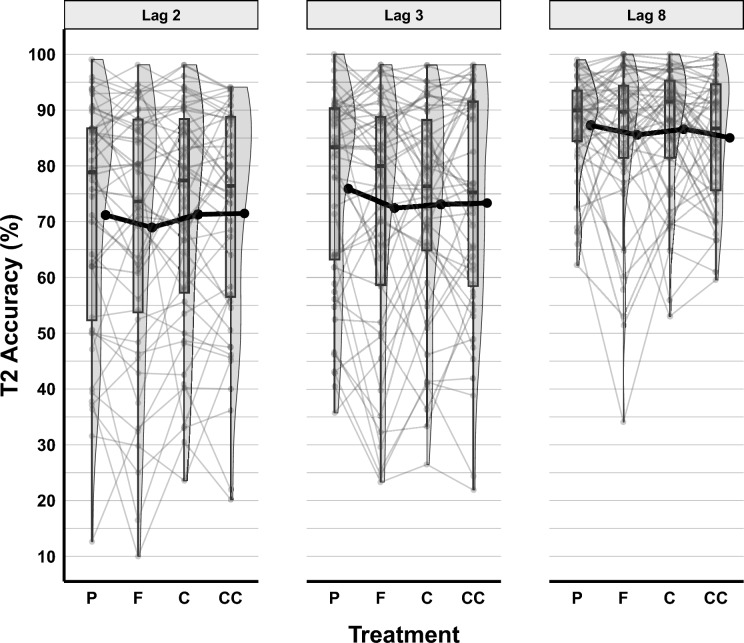
T2|T1 Accuracy in the RSVP Task across Treatment and Lag. Figure conventions follow Fig. 2

For T2|T1 accuracy, the GLMM showed that adding the random intercepts for subjects decreased deviation significantly [χ^2^_∆d_ = 5701.7, *df* = 1, *p* < 0.001, *BF*_*10*_ > 100]. Following this, the fixed effects were included in the model, and Lag [χ^2^_∆d_ = 969.99, *df* = 2, *p* < 0.001, *BF*_*10*_ > 100] improved the model significantly, but Treatment did not [χ^2^_∆d_ = 5.72, *df* = 3, *p* = 0.126, *BF*_*10*_ < 0.001]. Random slopes for fixed effects were also tested, and the model was better after adding the random slope to Lag [χ^2^_∆d_ = 677.72, *df* = 5, *p* < 0.001, *BF*_*10*_ > 100]. In the post-hoc analyses, neither the gender of participants [χ^2^_∆d_ = 0.46, *df* = 1,* p* = 0.499, *BF*_*10*_ = 0.005], nor their BMI scores [χ^2^_∆d_ = 0.011, *df* = 1,* p* = 0.918, *BF*_*10*_ = 0.004] improved the final model (see Table S3). T2|T1 accuracy across Lag and Treatment is shown in Fig. 3.

Tukey pairwise comparisons between Lag conditions showed that T2|T1 accuracy at Lag 8 (*prob* = 0.904, SE = 0.011, 95% CI [0.880–0.924]) was significantly higher than at Lag 3 (*prob* = 0.836, SE = 0.021, 95% CI [0.789–0.873]), *Z* = 5.80, *p* < 0.001, and higher than at Lag 2 (*prob* = 0.815, SE = 0.025, 95% CI [0.762–0.858]), *Z* = 6.33, *p* < 0.001. Also, at Lag 3, T2|T1 accuracy was significantly higher than at Lag 2, *Z* = 2.66, *p* = 0.021. These outcomes confirmed the presence of an attentional blink at the shorter lags.

**Fig. 4:**
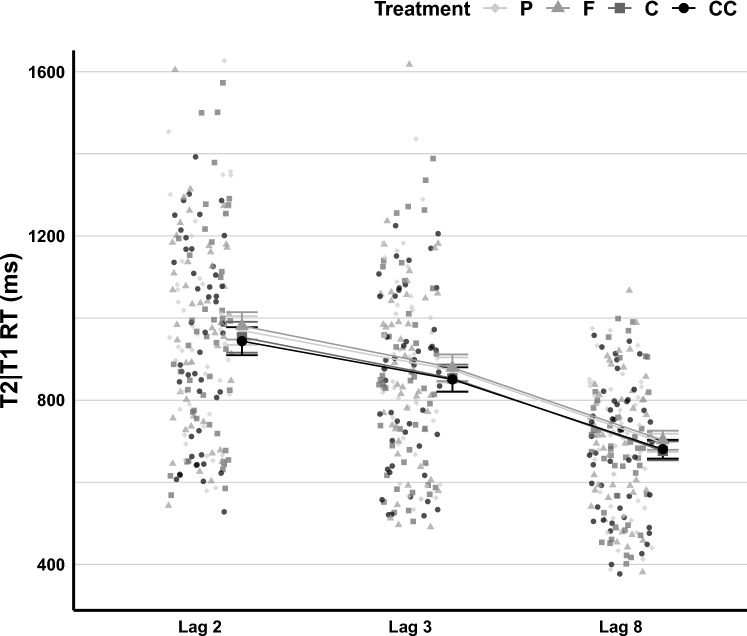
T2|T1 RT in the RSVP Task across Treatment and Lag. The figure illustrates individual RT means across various treatment conditions. The Placebo (P) condition is represented by light grey filled diamonds for both individual data points and larger dots indicating means and standard errors. The cocoa flavanols (F) condition is signified by medium-light grey filled triangles. The caffeine (C) condition is depicted using medium-dark grey filled squares. Lastly, the concurrent (CC) condition, which combined cocoa flavanols and caffeine, is represented by black filled circles

In the LMM on T2|T1 RT, deviance in the null model (without fixed and random effects) was significantly reduced [χ^2^_∆d_ = 17,466.0, *df* = 1, *p* < 0.001, *BF*_*10*_ > 100]. The fixed effects of Lag [χ^2^_∆d_ = 9596.3, *df* = 2, *p* < 0.001, *BF*_*10*_ > 100], and Treatment [χ^2^_∆d_ = 71.51, *df* = 3, *p* < 0.001, *BF*_*10*_ > 100] improved the model significantly, but the interaction of Lag and Treatment did not [χ^2^_∆d_ = 3.96, *df* = 6, *p* = 0.682, *BF*_*10*_ < 0.001]. Following the fixed effects, random slopes for Lag [χ^2^_∆d_ = 1989.3, *df* = 5, *p* < 0.001, *BF*_*10*_ > 100] were included in the model and improved it significantly, but random slopes for Treatment did not [χ^2^_∆d_ = 29.08, *df* = 24, *p* = 0.217, *BF*_*10*_ < 0.001]. In the exploratory analyses, neither the gender of participants [χ^2^_∆d_ = 9.17, *df* = 1,* p* = 0.003, *BF*_*10*_ = 0.467], nor their BMI scores [χ^2^_∆d_ = 6.28, *df* = 1,* p* = 0.012, *BF*_*10*_ = 0.110] improved the final model (Table S4). T2|T1 RT across Lag and Treatment is shown in Fig. 4.

Pairwise Tukey comparisons between Lag conditions showed that T2|T1 RT at Lag 8 (*Estimated marginal mean [EMM]* = 688, SE = 20.8, 95% CI [647–729]) was significantly lower than at Lag 3 (*EMM* = 863, SE = 29.2, 95% CI [806–921]), *Z* = 14.17, *p* < 0.001, and lower than at Lag 2 (*EMM* = 961, SE = 32.8, 95% CI [897–1025]), *Z* = 16.02, *p* < 0.001. Also, at Lag 3, T2|T1 RT was significantly lower than at Lag 2, *Z* = 12.62, *p* < 0.001. These RT results thus mirrored the typical accuracy pattern during the attentional blink.

LMM results showed that both the caffeine Treatment, (*b* = − 25.26, SE = 10.86, 95% CI [− 46.54–− 3.98],* t* = − 2.33, *p* = 0.020), and the concurrent flavanols and caffeine Treatment (*b* = − 24.97, SE = 10.84, 95% CI [− 46.22–− 3.72],* t* = -2.30, *p* = 0.021) significantly predicted T2|1 RT, but the cocoa flavanols Treatment did not (*b* = − 2.10, SE = 10.88, 95% CI [− 23.43–19.22],* t* = − 0.19, *p* = 0.847).

**Fig. 5:**
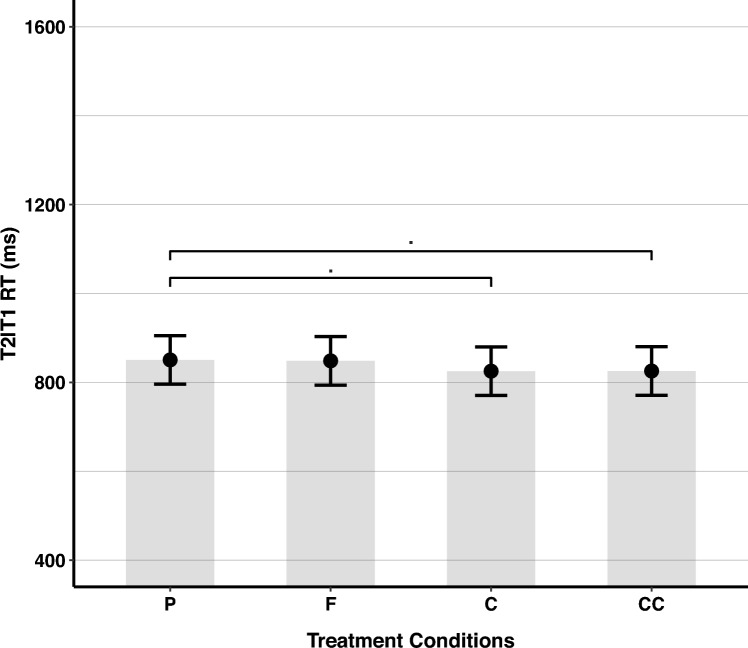
T2|T1 RT in the RSVP Task for Treatment, collapsed across Lag. The figure illustrates RT means for the different treatment conditions, collapsed across Lag. P refers to the placebo condition, F to cocoa flavanols, C to caffeine, and CC to the concurrent condition (flavanols and caffeine combined). p < .10

Based on Tukey pairwise comparisons (see Fig. 5), there was a marginally significant difference between the placebo (*EMM* = 851, SE = 27.9, 95% CI [796–905]) and caffeine (*EMM* = 825, SE = 27.9, 95% CI [771–880]) conditions, *Z* = 2.33, *p* = 0.092, and between the placebo and the concurrent flavanols and caffeine conditions (*EMM* = 826, SE = 27.9, 95% CI [771–880]), *Z* = 2.30, *p* = 0.097. However, T2|T1 RT in the cocoa flavanols condition (*EMM* = 848, SE = 27.9, 95% CI [794–903]) was not significantly different from the placebo condition, *Z* = 0.19, *p* = 0.997, from the caffeine condition,* Z* = − 2.14, *p* = 0.142, and from the concurrent flavanols and caffeine condition,* Z* = 2.10, *p* = 0.152. Also, in the caffeine condition, T2|T1 RT was not significantly different from that in the concurrent flavanols and caffeine condition,* Z* = − 0.03, *p* = 1.

### Spatial attention

**Fig. 6:**
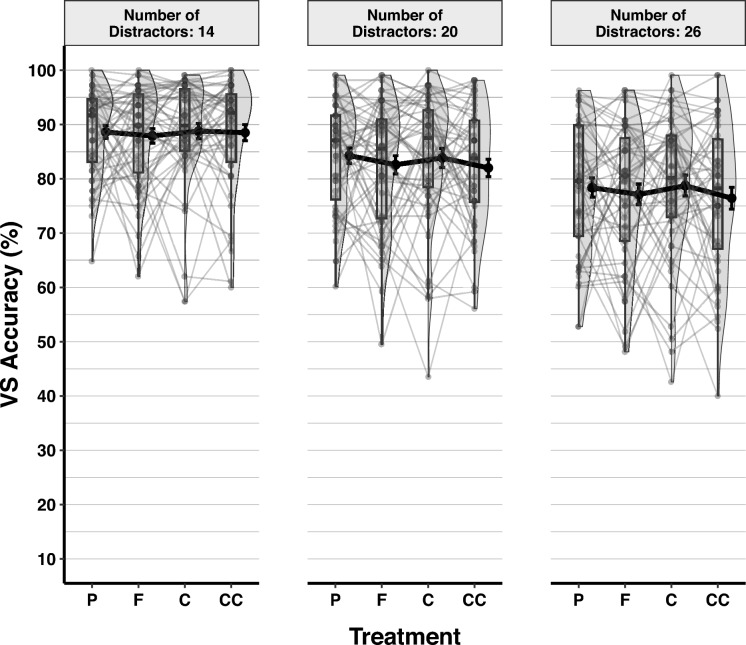
Accuracy in Visual Search by Treatment and Number of Distractors. Figure conventions follow Fig. 2

The GLMM showed that adding the random intercepts for subjects to the null model significantly decreased deviance [χ^2^_∆d_ = 2819.9, *df* = 1, *p* < 0.001, *BF*_*10*_ > 100]. Adding the number of Distractors as a fixed effect improved the model significantly [χ^2^_∆d_ = 912.62, *df* = 2, *p* < 0.001, *BF*_*10*_ > 100], but adding Treatment did not [χ^2^_∆d_ = 24.6, *df* = 3, *p* < 0.001, *BF*_*10*_ = 0.014]. Following the fixed effects, random slopes for the number of Distractors were added, but this did not improve the model significantly [χ^2^_∆d_ = 7.38, *df* = 5, *p* = 0.194, *BF*_*10*_ < 0.001]. Exploratory analyses showed that neither the gender of participants [χ^2^_∆d_ = 1.30, *df* = 1,* p* = 0.255, *BF*_*10*_ = 0.008], nor their BMI scores [χ^2^_∆d_ = 1.45, *df* = 1,* p* = 0.229, *BF*_*10*_ = 0.008] predicted visual search accuracy (Table S5). Visual search accuracy as a function of the number of Distractors and Treatment is shown in Fig. 6.

Tukey pairwise comparison results showed that as the number of Distractors increased, visual search accuracy decreased gradually. Search accuracy was significantly higher when 14 distractors were displayed (*prob* = 0.908, SE = 0.009, 95% CI [0.890–0.924]), than when 20 distractors (*prob* = 0.861, SE = 0.012, 95% CI [0.835–0.883]), *Z* = 15.99, *p* < 0.001, or 26 distractors (*prob* = 0.808, SE = 0.016, 95% CI [0.775–0.837]) were presented, *Z* = 30.14, *p* < 0.001. Additionally, when 20 distractors were shown, search accuracy was significantly higher than when 26 distractors were displayed, *Z* = 14.77, *p* < 0.001.

**Fig. 7:**
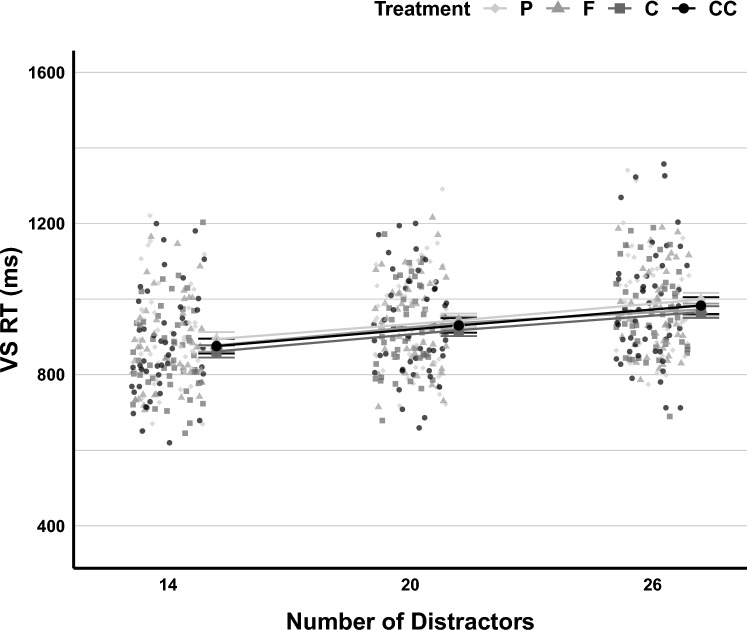
Visual Search RT across Treatment and Number of Distractors. Figure conventions follow Fig. 4

In the LMM analysis of visual search RT, including random intercepts for subjects in the model significantly reduced deviance [χ^2^_∆d_ = 5489.4, *df* = 1, *p* < 0.001, *BF*_*10*_ > 100]. Adding fixed effects of the number of Distractors [χ^2^_∆d_ = 1170.2, *df* = 2, *p* < 0.001, *BF*_*10*_ > 100] and Treatment [χ^2^_∆d_ = 67.37, *df* = 3, *p* < 0.001, *BF*_*10*_ > 100] also improved the model significantly. However, the interaction of the number of Distractors and Treatment conditions did not [χ^2^_∆d_ = 5.69, *df* = 6, *p* = 0.459, *BF*_*10*_ < 0.001]. Additionally, as random slopes, neither the number of Distractors [χ^2^_∆d_ = 23.08, *df* = 5, *p* = 0.0003, *BF*_*10*_ < 0.001] nor Treatment [χ^2^_∆d_ = 33.63, *df* = 18, *p* = 0.014, *BF*_*10*_ < 0.001] improved the model. In the exploratory analysis, neither the gender of participants [χ^2^_∆d_ = 8.81, *df* = 1,* p* = 0.003, *BF*_*10*_ = 0.360], nor their BMI scores [χ^2^_∆d_ = 6.08, *df* = 1,* p* = 0.014, *BF*_*10*_ = 0.091] improved the model (Table S6). Visual search RT by the number of Distractors and Treatment is shown in Fig. 7.

Tukey pairwise comparisons showed that visual search RT was significantly lower when 14 distractors (*EMM* = 876, SE = 14.6, 95% CI [848–905]) were presented than when 20 distractors (*EMM* = 932, SE = 14.6, 95% CI [904–961]), *Z* = 19.71, *p* < 0.001, or 26 distractors were displayed (*EMM* = 981, SE = 14.6, 95% CI [952–1009]), *Z* = 35.91, *p* < 0.001. Also, RT was significantly lower when 20 distractors were shown than when 26 distractors were shown, *Z* = 16.33, *p* < 0.001.

The caffeine Treatment condition predicted visual search RT significantly (*b* = − 16.42, SE = 7.60, 95% CI [− 31.31–− 1.53], *t* = − 2.16, *p* = 0.031), but the cocoa flavanols (*b* = − 6.53, SE = 7.57, 95% CI [− 21.37–8.31], *t* = − 0.86, *p* = 0.388) and concurrent conditions (*b* = − 12.34, SE = 7.66, 95% CI [− 27.36–2.68], *t* = − 1.61, *p* = 0.107) did not.

**Fig. 8:**
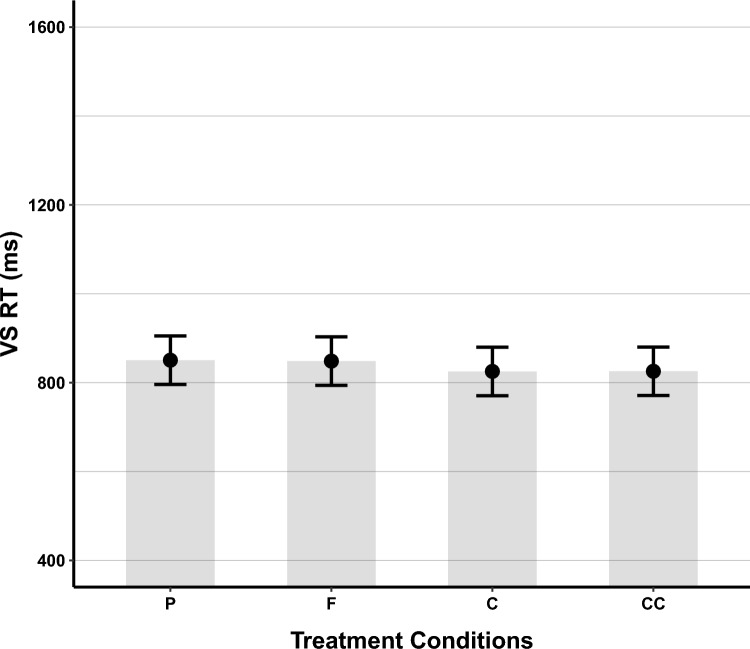
Visual Search RT, collapsed across the Number of Distractors. Figure conventions follow Fig. 5

Tukey test results (see Fig. 8) showed that visual search RT in the placebo condition (*EMM* = 939, SE = 15.3, 95% CI [909–969]) was not significantly different from the caffeine (*EMM* = 922, SE = 15.3, 95% CI [892–952]), *Z* = 2.16, *p* = 0.134, the cocoa flavanols (*EMM* = 932, SE = 15.3, 95% CI [902–962]), *Z* = 0.86, *p* = 0.824, or the concurrent condition (*EMM* = 926, SE = 15.3, 95% CI [896–956]), *Z* = 1.11, *p* = 0.373. Additionally, RT was not significantly different between the cocoa flavanols condition and the caffeine, *Z* = 1.30, *p* = 0.566, and concurrent condition, *Z* = 0.76, *p* = 0.871. There were also no significant differences between the caffeine and the concurrent conditions, *Z* = 0.54, *p* = 0.950.

### Visual working memory maintenance

**Fig. 9:**
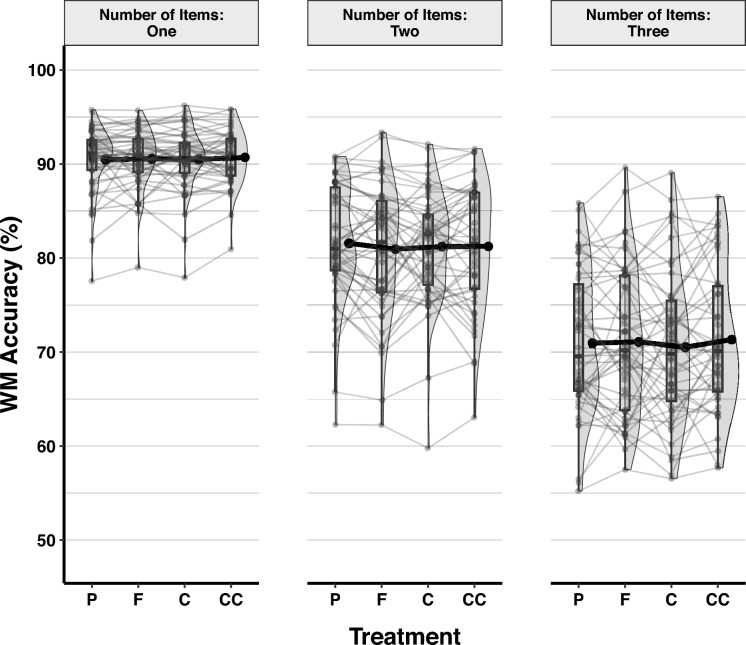
Response Accuracy in the Visual Working Memory Task by Treatment and Number of Items. Figure conventions follow Fig. 2

The LMM analysis of visual working memory accuracy showed that adding random intercepts for subjects to the null model decreased deviance significantly [χ^2^_∆d_ = 2900.6, *df* = 1, *p* < 0.001, *BF*_*10*_ > 100]. As fixed effect, the number of Items was added to the model, improving it significantly [χ^2^_∆d_ = 8403.0, *df* = 2,* p* < 0.001, *BF*_*10*_ > 100], but Treatment did not [χ^2^_∆d_ = 2.46, *df* = 3, *p* = 0.482, *BF*_*10*_ < 0.001]. Including the random slopes of the number of Items also improved the model significantly [χ^2^_∆d_ = 596.64, *df* = 5, *p* < 0.001, *BF*_*10*_ > 100]. Lastly, in the exploratory analyses, neither the gender of the participants [χ^2^_∆d_ = 6.32, *df* = 1,* p* = 0.012, *BF*_*10*_ = 0.157], nor their BMI scores [χ^2^_∆d_ = 0.03, *df* = 1,* p* = 0.869, *BF*_*10*_ = 0.0011] predicted visual working memory maintenance accuracy (Table S7). Response accuracy by the number of Items and Treatment is shown in Fig. 9.

Tukey pairwise comparisons between the number of Items showed that visual working memory maintenance accuracy was significantly higher when one item was presented (*EMM* = 90.6, SE = 0.44, 95% CI [89.7–91.4]), than when two items (*EMM* = 81.2, SE = 0.88, 95% CI [79.5–83.5]), *Z* = 16.74, *p* < 0.001, or three items were shown (*EMM* = 71.0, SE = 1.04, 95% CI [68.9–73.0]), *Z* = 25.57, *p* < 0.001. Furthermore, working memory maintenance accuracy was significantly higher in the two items condition than in the three items condition,* Z* = 25.01, *p* < 0.001.

## Discussion

We examined the acute effects of concurrently ingesting moderate doses of cocoa flavanols and caffeine on temporal and spatial attention, as well as working memory maintenance. The outcome can be easily summarized: We found no evidence for any effect related to these substances, apart from a marginal trend towards shorter T2 RT in RSVP due to the ingestion of caffeine. It is important to place these findings in the context of the other variables we manipulated. In the RSVP task, we manipulated the lag between targets, expecting to observe the attentional blink deficit and increased RTs at shorter lags. In the visual search task, we manipulated the number of simultaneous distractors, expecting increasing search RT when more distractors were shown. Finally, in the delayed recall task, we manipulated the number of items to be retained, expecting that more items would reduce recall performance. All of these manipulations were clearly successful, and produced the expected effects. Thus, the lack of effects associated with flavanols and caffeine cannot be explained by a failure to properly test attention and working memory.

In a similar vein, there were no indications that the tasks we presently tested led to performance that was close to a bottom or ceiling level that might have obscured treatment effects, especially in the most critical and difficult conditions (e.g., short lag in RSVP). Whereas there was substantial intra- and inter-individual variance in the data, despite the relative homogeneity of our tested sample (i.e., local university students), the resultant treatment means were nevertheless very close to each other, suggesting that this variance did not hide differences that might have proven reliable with a larger sample.

It might be concluded that the physiological effects of cocoa flavanols and caffeine do not result in changes at the cognitive level. In other words, assuming that these effects indeed took place in our study, perhaps increased cerebral blood flow, enhanced synaptic plasticity, and a release from adenosine-related inhibition in the release of neurotransmitters, simply do not help attention in time and space, or working memory. There could be various reasons for why this might be the case, but one might speculate that the bottlenecks in these cognitive functions just do not lie in these particular physiological conditions.

Another possible explanation for the lack of acute effects could be the dosages that we presently administered, which were 415 mg of cocoa flavanols and 215 mg of caffeine. However, there is little reason to suspect that these dosages would be ineffective. With regard to flavanols, dosages in the range of 83–994 mg have produced acute effects in previous studies [24, 27]. Similarly, with regard to caffeine, dosages in the range of 60–450 mg have been considered as effective previously, particularly on attention-related functions [72], with limited evidence for dose-dependency within this range. More recently, a dose-dependent range between 40 and 300 mg has been identified as effective in improving attentional, and less consistently, memory-related processes in well-rested individuals [5]. Thus, although there is some variation in the literature, and although we did not systematically test different dosage levels in the present study, the chosen amounts seem appropriate to elicit acute cognitive effects.

Perhaps the most likely account for the current null findings is that the cognitive functions presently tested are not the ones that were critically taxed in previous positive reports. The kind of tasks that are frequently used to assess psychopharmacological effects tend to combine (and as we have argued, conflate) different cognitive functions. Thereby, an effect on one of those may produce an overall effect that implicates also other functions, even though those are in fact not modulated. Case in point might be the N-back task (e.g., [28]), which arguably taxes not only working memory, but also requires active inhibition of currently irrelevant and previously relevant items (cf. [57]). It is conceivable that although the N-back task is often interpreted as a working memory task, actual acute effects of cocoa flavanols and/or caffeine might be on the inhibitory part, even if it is a lesser aspect of this task.

This account would also fit to previous findings from our own lab, in which we found no acute effect of cocoa flavanols on (pure) working memory either [29]. It would also fit to another previous study on the effects of cocoa flavanols on attention, in which we observed a positive effect on RT in visual search, contrary to the present findings [25]. Although surprising at first glance, the visual search task used by Karabay and colleagues was of a special kind, in which a salient second singleton stimulus appeared next to target stimuli in the search arrays (cf. [82]). It might have been the need to inhibit this second singleton that was facilitated acutely by the ingestion of cocoa flavanols in that study. In the present study, this inhibitory aspect was lacking from the more typical visual search task we used, even though overall the present task was at least as difficult.

## Limitations and recommendations for future research

Our study has some limitations that might be addressed in future studies on the acute effects of caffeine and cocoa flavanols. For instance, these might focus on different cognitive functions that the ones tested presently. Executive and inhibitory functions, such as tested in Stroop or Flanker tasks (e.g., [26, 32, 33, 47]), may prove more sensitive to these substances, where attention and working memory did not. As previous research observed acute effects on tasks in which cognitive functions are combined (e.g., [24, 41]), an obvious choice would be to revert to such tasks. However, we would argue that it is important to then devise a way to systematically vary the degree to which different cognitive functions are taxed, so that the locus of the effects can then be determined. It might also be worthwhile to test different dosage levels, as dosage-response curves may yield surprising maxima. Furthermore, it might be advisable to target specific groups that perform at a lower baseline level than our healthy young university students did, such as elderly, fatigued, or sleep-deprived individuals. Finally, we did not strictly control the (habitual) intake of flavanols and caffeine, which would also be advisable to assess possible contributions of withdrawal effects, which has been a concern in the caffeine literature in particular (e.g., [48],but see also [72]).

## Conclusion

We found no evidence for any acute effect of the ingestion of commonly tested doses of cocoa flavanols and caffeine, nor of their concurrent consumption, on temporal attention, spatial attention, and working memory, in our sample. Possibly, flavanols and caffeine mainly affect early visual functions, or other cognitive ones, such as executive functions, which may account for previously observed enhancements.

## Supplementary Information

Below is the link to the electronic supplementary material.

Supplementary file1 (DOCX 48 KB)

## Data Availability

The data of the study and analysis codes, including plots, are available on the Open Science Framework, at: https://osf.io/kjgyd.

## Footnotes

1 The focus of the current work is on acute cognitive effects. See Socci et al. [1, 2, 3] Karataş et al. [4], and McLellan et al. [5] for reviews that also include long-term effects.

## References

[1] Socci V, Tempesta D, Desideri G et al (2017) Enhancing human cognition with cocoa flavonoids. Front Nutr 4:19. 10.3389/fnut.2017.00019 28560212 10.3389/fnut.2017.00019 PMC5432604

[2] Haskell-Ramsay CF, Jackson PA, Forster JS et al (2018) The acute effects of caffeinated black coffee on cognition and mood in healthy young and older adults. Nutrients 10:1386. 10.3390/nu10101386 30274327 10.3390/nu10101386 PMC6213082

[3] Haskell-Ramsay CF, Schmitt J, Actis-Goretta L (2018) The impact of epicatechin on human cognition: the role of cerebral blood flow. Nutrients 10:986. 10.3390/nu10080986 30060538 10.3390/nu10080986 PMC6115745

[4] Karataş, O., Karabay, A., & Alıcı, T. (2022). Uzun süreli kakao flavanolleri alımının bilişsel işlevlere ve duygudurumuna etkileri ve bu etkilerin altındaki fizyolojik mekanizmalar: Bir derleme çalışması. *Türk Psikoloji Yazıları, 25*, 1–26. 10.31828/tpy1301996120211203m000043

[5] McLellan TM, Caldwell JA, Lieberman HR (2016) A review of caffeine’s effects on cognitive, physical and occupational performance. Neurosci Biobehav Rev 71:294–312. 10.1016/j.neubiorev.2016.09.001 27612937 10.1016/j.neubiorev.2016.09.001

[6] Fisher ND, Hughes M, Gerhard-Herman M, Hollenberg NK (2003) Flavanol-rich cocoa induces nitric-oxide-dependent vasodilation in healthy humans. J Hypertens 21:2281–2286. 10.1097/00004872-200312000-00016 14654748 10.1097/00004872-200312000-00016

[7] Fraga CG, Litterio MC, Prince PD, Calabró V, Piotrkowski B, Galleano M (2010) Cocoa flavanols: effects on vascular nitric oxide and blood pressure. J Clin Biochem Nutrit 48:63–67. 10.3164/jcbn.11-010fr 21297914 10.3164/jcbn.11-010FR PMC3022066

[8] Calver A, Collier J, Vallance P (1992) Nitric oxide and blood vessels: physiological role and clinical implications. Biochem Educ 20:130–135. 10.1016/0307-4412(92)90048-q

[9] Garthwaite J (1991) Glutamate, nitric oxide and cell-cell signalling in the nervous system. Trends Neurosci 14:60–67. 10.1016/0166-2236(91)90022-m 1708538 10.1016/0166-2236(91)90022-m

[10] Spencer JPE (2007) The interactions of flavonoids within neuronal signalling pathways. Genes Nutr 2:257–273. 10.1007/s12263-007-0056-z 18850181 10.1007/s12263-007-0056-z PMC2474943

[11] Vincent SR (2010) Nitric oxide neurons and neurotransmission. Prog Neurobiol 90:246–255. 10.1016/j.pneurobio.2009.10.007 19853011 10.1016/j.pneurobio.2009.10.007

[12] Epstein FH, Moncada S, Higgs A (1993) The L-arginine-nitric oxide pathway. N Engl J Med 329:2002–2012. 10.1056/nejm199312303292706 7504210 10.1056/NEJM199312303292706

[13] Huang EP (1997) Synaptic plasticity: a role for nitric oxide in LTP. Curr Biol 7:R141–R143. 10.1016/s0960-9822(97)70073-3 9162474 10.1016/s0960-9822(97)70073-3

[14] Vaccaro MG, Innocenti B, Cione E et al (2024) Acute effects of a chewable beetroot-based supplement on cognitive performance: a double-blind randomized placebo-controlled crossover clinical trial. Eur J Nutr 63:303–321 37875637 10.1007/s00394-023-03265-y PMC10799154

[15] Fredholm BB (1979) Are methylxanthine effects due to antagonism of endogenous adenosine? Trends Pharmacol Sci 1:129–132. 10.1016/0165-6147(79)90046-4

[16] Flagmeyer I, Haas HL, Stevens DR (1997) Adenosine A1 receptor-mediated depression of corticostriatal and thalamostriatal glutamatergic synaptic potentials in vitro. Brain Res 778:178–185. 10.1016/S0006-8993(97)01060-3 9462890 10.1016/s0006-8993(97)01060-3

[17] Okada M, Kawata Y, Murakami T et al (1999) Differential effects of adenosine receptor subtypes on release and reuptake of hippocampal serotonin. Eur J Neurosci 11:1–9. 10.1046/j.1460-9568.1999.00415.x 9987006 10.1046/j.1460-9568.1999.00415.x

[18] Ferré S (2010) Role of the central ascending neurotransmitter systems in the psychostimulant effects of caffeine. J Alzheimers Dis 20:S35–S49. 10.3233/JAD-2010-1400 20182056 10.3233/JAD-2010-1400 PMC9361505

[19] Field DT, Williams CM, Butler LT (2011) Consumption of cocoa flavanols results in an acute improvement in visual and cognitive functions. Physiol Behav 103:255–260. 10.1016/j.physbeh.2011.02.013 21324330 10.1016/j.physbeh.2011.02.013

[20] Puell MC, de Pascual-Teresa S (2021) The acute effect of cocoa and red-berries on visual acuity and cone-mediated dark adaptation in healthy eyes. Journal of Functional Foods 81:104435. 10.1016/j.jff.2021.104435

[21] Rabin JC, Karunathilake N, Patrizi K (2018) Effects of milk vs dark chocolate consumption on visual acuity and contrast sensitivity within 2 hours: a randomized clinical trial. JAMA Ophthalmology 136:678–681. 10.1001/jamaophthalmol.2018.0978 29710322 10.1001/jamaophthalmol.2018.0978 PMC6145773

[22] Siedlecki J, Mohr N, Luft N et al (2019) Effects of flavanol-rich dark chocolate on visual function and retinal perfusion measured with optical coherence tomography angiography: a randomized clinical trial. JAMA Ophthalmol 137:1373–1379. 10.1001/jamaophthalmol.2019.3731 31556937 10.1001/jamaophthalmol.2019.3731 PMC6763974

[23] Boolani A, Lindheimer JB, Loy BD, Crozier S, O’Connor PJ (2017) Acute effects of brewed cocoa consumption on attention, motivation to perform cognitive work and feelings of anxiety, energy and fatigue: a randomised, placebo-controlled crossover experiment. BMC Nutrition 3:1–11. 10.1186/s40795-016-0117-z

[24] Scholey AB, French SJ, Morris PJ, Kennedy DO, Milne AL, Haskell CF (2010) Consumption of cocoa flavanols results in acute improvements in mood and cognitive performance during sustained mental effort. J Psychopharmacol 24:1505–1514. 10.1177/0269881109106923 19942640 10.1177/0269881109106923

[25] Karabay A, Saija JD, Field DT, Akyürek EG (2018) The acute effects of cocoa flavanols on temporal and spatial attention. Psychopharmacology 235:1497–1511. 10.1007/s00213-018-4861-4 29502273 10.1007/s00213-018-4861-4 PMC5920121

[26] Massee LA, Ried K, Pase M et al (2015) The acute and subchronic effects of cocoa flavanols on mood, cognitive and cardiovascular health in young healthy adults: a randomized, controlled trial. Front Pharmacol 6:00093. 10.3389/fphar.2015.00093 10.3389/fphar.2015.00093 PMC4438591 26042037

[27] Lamport DJ, Christodoulou E, Achilleos C (2020) Beneficial effects of dark chocolate for episodic memory in healthy young adults: a parallel-groups acute intervention with a white chocolate control. Nutrients 21:483. 10.3390/nu12020483 10.3390/nu12020483 PMC7071338 32075015

[28] Grassi D, Socci V, Tempesta D et al (2016) Flavanol-rich chocolate acutely improves arterial function and working memory performance counteracting the effects of sleep deprivation in healthy individuals. J Hypertens 34:1298–1308. 10.1097/HJH.0000000000000926 27088635 10.1097/HJH.0000000000000926

[29] Altınok A, Karabay A, Akyürek EG (2022) Acute effects of cocoa flavanols on visual working memory: Maintenance and updating. Eur J Nutr 61:1665–1678. 10.1007/s00394-021-02767-x 35031887 10.1007/s00394-021-02767-x

[30] Crews WD, Harrison DW, Wright JW (2008) A double-blind, placebo-controlled, randomized trial of the effects of dark chocolate and cocoa on variables associated with neuropsychological functioning and cardiovascular health: clinical findings from a sample of healthy, cognitively intact older adults. Am J Clin Nutr 87:872–880. 10.1093/ajcn/87.4.872 18400709 10.1093/ajcn/87.4.872

[31] Pase MP, Scholey AB, Pipingas A et al (2013) Cocoa polyphenols enhance positive mood states but not cognitive performance: a randomized, placebo-controlled trial. J Psychopharmacol 27:451–458. 10.1177/0269881112473791 23364814 10.1177/0269881112473791

[32] Tsukamoto H, Suga T, Ishibashi A et al (2018) Flavanol-rich cocoa consumption enhances exercise-induced executive function improvements in humans. Nutrition 46:90–96. 10.1016/j.nut.2017.08.017 29290363 10.1016/j.nut.2017.08.017

[33] Decroix L, Tonoli C, Soares DD et al (2016) Acute cocoa flavanol improves cerebral oxygenation without enhancing executive function at rest or after exercise. Appl Physiol Nutr Metab 41:1225–1232. 10.1139/apnm-2016-0245 27849355 10.1139/apnm-2016-0245

[34] Francis ST, Head K, Morris PG, Macdonald IA (2006) The effect of flavanol-rich cocoa on the fMRI response to a cognitive task in healthy young people. J Cardiovasc Pharmacol 47:215–220. 10.1097/00005344-200606001-00018 10.1097/00005344-200606001-00018 16794461

[35] Diamond AL, Cole RE (1970) Visual threshold as a function of test area and caffeine administration. Psychonomic Science 20:109–111. 10.3758/BF03335627

[36] Kravkov SV (1939) The influence of caffeine on the color sensitivity. Acta Ophthalmol 17:89–94. 10.1111/j.1755-3768.1939.tb04309.x

[37] Nguyen BN, Hew S-A, Ly J et al (2017) Acute caffeine ingestion affects surround suppression of perceived contrast. J Psychopharmacol 32:81–88. 10.1177/0269881117725684 28879800 10.1177/0269881117725684

[38] Redondo B, Jiménez R, Molina R, Dalton K, Vera J (2021) Effects of caffeine ingestion on dynamic visual acuity: a placebo-controlled, double-blind, balanced-crossover study in low caffeine consumers. Psychopharmacology 238:3391–3398. 10.1007/s00213-021-05953-1 34420061 10.1007/s00213-021-05953-1 PMC8629887

[39] Adan A, Serra-Grabulosa JM (2010) Effects of caffeine and glucose, alone and combined, on cognitive performance. Hum Psychopharmacol 25:310–317. 10.1002/hup.1115 20521321 10.1002/hup.1115

[40] Giles GE, Mahoney CR, Brunyé TT et al (2012) Differential cognitive effects of energy drink ingredients: Caffeine, taurine, and glucose. Pharmacol Biochem Behav 102:569–577. 10.1016/j.pbb.2012.07.004 22819803 10.1016/j.pbb.2012.07.004

[41] Haskell CF, Kennedy DO, Wesnes KA, Scholey AB (2005) Cognitive and mood improvements of caffeine in habitual consumers and habitual non-consumers of caffeine. Psychopharmacology 179:813–825. 10.1007/s00213-004-2104-3 15678363 10.1007/s00213-004-2104-3

[42] Smit HJ, Rogers PJ (2000). Effects of low doses of caffeine on cognitive performance, mood and thirst in low and higher caffeine consumers. Psychopharmacology. 152:167–173. 10.1007/s002130000506 10.1007/s002130000506 11057520

[43] Hasenfratz M, Bättig K (1994) Acute dose-effect relationships of caffeine and mental performance, EEG, cardiovascular and subjective parameters. Psychopharmacology 114:281–287. 10.1007/BF02244850 7838921 10.1007/BF02244850

[44] Haskell CF, Kennedy DO, Milne AL, Wesnes KA, Scholey AB (2008) The effects of _L_-theanine, caffeine and their combination on cognition and mood. Biol Psychol 77:113–122. 10.1016/j.biopsycho.2007.09.008 18006208 10.1016/j.biopsycho.2007.09.008

[45] Warburton DM (1995) Effects of caffeine on cognition and mood without caffeine abstinence. Psychopharmacology 119:66–70. 10.1007/BF02246055 7675951 10.1007/BF02246055

[46] Lorist MM, Snel J, Kok A, Mulder G (1996) Acute effects of caffeine on selective attention and visual search processes. Psychophysiology 33:354–361. 10.1111/j.1469-8986.1996.tb01059.x 8753934 10.1111/j.1469-8986.1996.tb01059.x

[47] Kenemans JL, Verbaten MN (1998) Caffeine and visuo-spatial attention. Psychopharmacology 135:353–360. 10.1007/s002130050522 9539259 10.1007/s002130050522

[48] Rogers PJ, Heatherley SV, Mullings EL, Smith JE (2013) Faster but not smarter: effects of caffeine and caffeine withdrawal on alertness and performance. Psychopharmacology 226:229–240. 10.1007/s00213-012-2889-4 23108937 10.1007/s00213-012-2889-4

[49] Childs E, de Wit H (2006) Subjective, behavioral, and physiological effects of acute caffeine in light, nondependent caffeine users. Psychopharmacology 185:514–523. 10.1007/s00213-006-0341-3 16541243 10.1007/s00213-006-0341-3

[50] Addicott MA, Laurienti PJ (2009) A comparison of the effects of caffeine following abstinence and normal caffeine use. Psychopharmacology 207:423–431. 10.1007/s00213-009-1668-3 19777214 10.1007/s00213-009-1668-3 PMC2941158

[51] Klaassen EB, de Groot RHM, Evers EAT et al (2013) The effect of caffeine on working memory load-related brain activation in middle-aged males. Neuropharmacology 64:160–167. 10.1016/j.neuropharm.2012.06.026 22728314 10.1016/j.neuropharm.2012.06.026

[52] Koppelstaetter F, Poeppel TD, Siedentopf CM et al (2008) Does caffeine modulate verbal working memory processes? An fMRI study. Neuroimage 39:492–499. 10.1016/j.neuroimage.2007.08.037 17936643 10.1016/j.neuroimage.2007.08.037

[53] Tieges Z, Snel J, Kok A et al (2006) Caffeine improves anticipatory processes in task switching. Biol Psychol 73:101–113. 10.1016/j.biopsycho.2005.12.005 16549227 10.1016/j.biopsycho.2005.12.005

[54] van den Berg B, de Jong M, Woldorff MG, Lorist MM (2021) Caffeine boosts preparatory attention for reward-related stimulus information. J Cogn Neurosci 33:104–118. 10.1162/jocn_a_01630 32985946 10.1162/jocn_a_01630

[55] Schuster J, Mitchell ES (2019) More than just caffeine: Psychopharmacology of methylxanthine interactions with plant-derived phytochemicals. Progress Neuropsychopharmacol Biol Psych 89:263–274. 10.1016/j.pnpbp.2018.09.005 10.1016/j.pnpbp.2018.09.005 30213684

[56] Ward-Ritacco CL, Wilson AR, O’Connor PJ (2021) An apple extract beverage combined with caffeine can improve alertness, mental fatigue, and information processing speed. Journal of Cognitive Enhancement 5:267–279. 10.1007/s41465-020-00204-1

[57] Jaeggi SM, Buschkuehl M, Perrig WJ, Meier B (2010) The concurrent validity of the N-back task as a working memory measure. Memory 18:394–412. 10.1080/09658211003702171 20408039 10.1080/09658211003702171

[58] Broadbent DE, Broadbent MHP (1987) From detection to identification: response to multiple targets in rapid serial visual presentation. Percept Psychophys 42:105–113. 10.3758/BF03210498 3627930 10.3758/bf03210498

[59] Raymond JE, Shapiro KL, Arnell KM (1992) Temporary suppression of visual processing in an RSVP task: an attentional blink? J Exp Psychol Hum Percept Perform 18:849–860. 10.1037/0096-1523.18.3.849 1500880 10.1037//0096-1523.18.3.849

[60] Martens S, Wyble B (2010) The attentional blink: Past, present, and future of a blind spot in perceptual awareness. Neurosci Biobehav Rev 34:947–957. 10.1016/j.neubiorev.2009.12.005 20025902 10.1016/j.neubiorev.2009.12.005 PMC2848898

[61] Olivers CNL, Meeter M (2008) A boost and bounce theory of temporal attention. Psychol Rev 115:836–863. 10.1037/a0013395 18954206 10.1037/a0013395

[62] Taatgen NA, Juvina I, Schipper M, Borst JP, Martens S (2009) Too much control can hurt: a threaded cognition model of the attentional blink. Cogn Psychol 59:1–29. 10.1016/j.cogpsych.2008.12.002 19217086 10.1016/j.cogpsych.2008.12.002

[63] Wyble B, Bowman H, Nieuwenstein M (2009) The attentional blink provides episodic distinctiveness: sparing at a cost. J Exp Psychol Hum Percept Perform 35:787–807. 10.1037/a0013902 19485692 10.1037/a0013902 PMC2743522

[64] Wolfe J, Horowitz T (2017) Five factors that guide attention in visual search. Nature Human Behavior 1:0058. 10.1038/s41562-017-0058 10.1038/s41562-017-0058 PMC9879335 36711068

[65] Downing PE (2000) Interactions between visual working memory and selective attention. Psychol Sci 11:467–473. 10.1111/1467-9280.00290 11202491 10.1111/1467-9280.00290

[66] Gazzaley A, Nobre AC (2012) Top-down modulation: bridging selective attention and working memory. Trends Cogn Sci 16:129–135. 10.1016/j.tics.2011.11.014 22209601 10.1016/j.tics.2011.11.014 PMC3510782

[67] McGabe DP (2008) The role of covert retrieval in working memory span tasks: Evidence from delayed recall tests. J Mem Lang 58:480–494. 10.1016/j.jml.2007.04.004 19633737 10.1016/j.jml.2007.04.004 PMC2715014

[68] Cowan N (2001) The magical number 4 in short-term memory: a reconsideration of mental storage capacity. Behav Brain Sci 24:87–114. 10.1017/S0140525X01003922 11515286 10.1017/s0140525x01003922

[69] Faul F, Erdfelder E, Lang AG, Buchneri A (2007) G*Power 3: A flexible statistical power analysis program for the social, behavioral, and biomedical sciences. Behaviour Research Methods 39:175–191. 10.3758/bf03193146 10.3758/bf03193146 17695343

[70] Mathôt, S., Schreij, D., Theeuwes, J. (2012) OpenSesame: An open-source, graphical experiment builder for the social sciences. Behavioral Research Methods. 44:314–324. 10.3758/s13428-011-0168-7 10.3758/s13428-011-0168-7 PMC3356517 22083660

[71] Lanini J, Galduróz JCF, Pompéia S (2016) Acute personalised habitual caffeine doses improve attention and have selective effects when considering the fractionation of executive functions. Hum Psychopharmacol Clin Exp 31:29–43. 10.1002/hup.2511 10.1002/hup.2511 26621326

[72] Einöther SJL, Giesbrecht T (2013) Caffeine as an attention enhancer: Reviewing existing assumptions. Psychopharmacology 225:251–274. 10.1007/s00213-012-2917-4 23241646 10.1007/s00213-012-2917-4

[73] McCusker RR, Fuehrlein B, Goldberger BA, Gold MS, Cone EJ (2006) Caffeine content of decaffeinated coffee. J Anal Toxicol 30:611–613. 10.1093/jat/30.8.611 17132260 10.1093/jat/30.8.611

[74] Beaumont M, Batejat D, Pierard C, Coste O, Doireau P, Van Beers P, Chauffard F, Chassard D, Enslen M, Denis JB, Lagarde D (2001) Slow release caffeine and prolonged (64-h) continuous wakefulness: effects on vigilance and cognitive performance. J Sleep Res 10:265–276. 10.1046/j.1365-2869.2001.00266.x 11903856 10.1046/j.1365-2869.2001.00266.x

[75] Lamport DJ, Pal D, Moutsiana C, Field DT, Williams CM, Spencer JPE, Butler LT (2015) The effect of flavanol-rich cocoa on cerebral perfusion in healthy older adults during conscious resting state: a placebo controlled, crossover, acute trial. Psychopharmacology 232:3227–3234. 10.1007/s00213-015-3972-4 26047963 10.1007/s00213-015-3972-4 PMC4534492

[76] Ruijter J, Lorist MM, Snel J, De Ruiter MB (2000) The influence of caffeine on sustained attention: an ERP study. Pharmacol Biochem Behav 66:29–37. 10.1016/S0091-3057(00)00229-X 10837841 10.1016/s0091-3057(00)00229-x

[77] R Core Team. (2018). *R: A language and environment for statistical computing* [Computer software]. https://www.r-project.org/

[78] Bates, D., Mächler, M., Bolker, B., & Walker, S. (2015). Fitting linear mixed-effects models using lme4. *Journal of Statistical Software, 67*, 1–48. 10.18637/jss.v067.i01

[79] Wickham, H. (2016). *ggplot2: Elegant graphics for data analysis. Springer-Verlag. *https://ggplot2.tidyverse.org

[80] Lüdecke, D. (2022). *sjPlot: Data visualization for statistics in social science* (R package version 2.8.11). https://cran.r-project.org/package=sjPlot

[81] Wagenmakers EJ (2007) A practical solution to the pervasive problems of p values. Psychon Bull Rev 14:779–804. 10.3758/BF03194105 18087943 10.3758/bf03194105

[82] Akyürek EG, Schubö A (2011) The allocation of attention in displays with simultaneously presented singletons. Biol Psychol 87:218–225. 10.1016/j.biopsycho.2011.02.022 21406208 10.1016/j.biopsycho.2011.02.022

