# Supplementary material for: Concurrent consumption of cocoa flavanols and caffeine does not acutely modulate working memory and attention
Source: Eur J Nutr. 2024 Nov 28;64(1):35. doi: 10.1007/s00394-024-03514-8 (PMC11604789; doi:10.1007/s00394-024-03514-8)
Supplement: Supplementary file 1 — Supplementary file1 (DOCX 48 KB) [file 394_2024_3514_MOESM1_ESM.docx]

# Supplementary Materials

Table S1

*Counter-balancing and Randomization List for Treatments and Tasks*

|  |  | | **Female** | | | | | | | | |
| --- | --- | --- | --- | --- | --- | --- | --- | --- | --- | --- | --- |
|  | ***Treatment Order*** | | | | | | |  | ***Task Order*** | | |
|  | ***Drink 1*** | | | ***Drink 2*** | ***Drink 3*** | | ***Drink 4*** |  | ***Task 1*** | ***Task 2*** | ***Task 3*** |
| 1 | CC | | | F | C | | P |  | RSVP | DR | VS |
| 2 | F | | | C | CC | | P |  | DR | VS | RSVP |
| 3 | CC | | | P | C | | F |  | VS | RSVP | DR |
| 4 | P | | | C | CC | | F |  | VS | DR | RSVP |
| 5 | C | | | CC | P | | F |  | VS | RSVP | DR |
| 6 | F | | | P | C | | CC |  | DR | VS | RSVP |
| 7 | P | | | F | CC | | C |  | DR | RSVP | VS |
| 8 | C | | | P | CC | | F |  | RSVP | DR | VS |
| 9 | C | | | F | CC | | P |  | VS | RSVP | DR |
| 10 | F | | | C | P | | CC |  | DR | VS | RSVP |
| 11 | C | | | CC | F | | P |  | RSVP | DR | VS |
| 12 | CC | | | F | P | | C |  | DR | RSVP | VS |
| 13 | F | | | CC | P | | C |  | DR | VS | RSVP |
| 14 | C | | | P | F | | CC |  | RSVP | VS | DR |
| 15 | P | | | F | C | | CC |  | VS | DR | RSVP |
| 16 | CC | | | C | P | | F |  | DR | RSVP | VS |
| 17 | CC | | | P | F | | C |  | RSVP | VS | DR |
| 18 | P | | | CC | C | | F |  | VS | RSVP | DR |
| 19 | F | | | CC | C | | P |  | RSVP | DR | VS |
| 20 | CC | | | C | F | | P |  | RSVP | VS | DR |
| 21 | C | | | F | P | | CC |  | DR | RSVP | VS |
| 22 | F | | | P | CC | | C |  | VS | DR | RSVP |
| 23 | P | | | C | F | | CC |  | VS | DR | RSVP |
| 24 | P | | | CC | F | | C |  | RSVP | VS | DR |
|  | | **Male** | | | | | | | | | |
|  | ***Treatment Order*** | | | | | | |  | ***Task Order*** | | |
|  | ***Drink 1*** | | | ***Drink 2*** | | ***Drink 3*** | ***Drink 4*** |  | ***Task 1*** | ***Task 2*** | ***Task 3*** |
| 1 | C | | | CC | | F | P |  | RSVP | DR | VS |
| 2 | CC | | | P | | C | F |  | DR | RSVP | VS |
| 3 | P | | | CC | | F | C |  | VS | RSVP | DR |
| 4 | CC | | | F | | C | P |  | RSVP | VS | DR |
| 5 | F | | | P | | C | CC |  | RSVP | DR | VS |
| 6 | P | | | F | | C | CC |  | VS | RSVP | DR |
| 7 | F | | | P | | CC | C |  | RSVP | VS | DR |
| 8 | C | | | P | | CC | F |  | VS | DR | RSVP |
| 9 | C | | | F | | CC | P |  | DR | VS | RSVP |
| 10 | CC | | | P | | F | C |  | VS | DR | RSVP |
| 11 | CC | | | C | | P | F |  | RSVP | VS | DR |
| 12 | CC | | | C | | F | P |  | VS | RSVP | DR |
| 13 | P | | | F | | CC | C |  | RSVP | DR | VS |
| 14 | F | | | CC | | C | P |  | DR | RSVP | VS |
| 15 | P | | | CC | | C | F |  | VS | DR | RSVP |
| 16 | F | | | CC | | P | C |  | RSVP | DR | VS |
| 17 | C | | | F | | P | CC |  | DR | RSVP | VS |
| 18 | F | | | C | | CC | P |  | DR | VS | RSVP |
| 19 | P | | | C | | F | CC |  | RSVP | VS | DR |
| 20 | P | | | C | | CC | F |  | DR | VS | RSVP |
| 21 | C | | | P | | F | CC |  | VS | DR | RSVP |
| 22 | C | | | CC | | P | F |  | VS | RSVP | DR |
| 23 | F | | | C | | P | CC |  | DR | VS | RSVP |
| 24 | CC | | | F | | P | C |  | DR | RSVP | VS |

*P: placebo, C: caffeine, F: cocoa flavanols, CC: concurrent caffeine and cocoa flavanols, RSVP: rapid serial visual presentation, DR: delayed recall, VS: visual search*

Table S2

*GLMM Results for the Effects of Flavanols and Caffeine on T1 Accuracy*

|  | **T1 Accuracy** | | | | |
| --- | --- | --- | --- | --- | --- |
| **Fixed Effects** | *Odds Ratios* | *std. Error* | *CI* | *z* | *p* |
| Intercept | 7.25 | 0.84 | 5.78 – 9.09 | 17.19 | **<0.001** |
| Lag [Lag 3] | 1.33 | 0.05 | 1.24 – 1.43 | 7.87 | **<0.001** |
| Lag [Lag 8] | 1.39 | 0.05 | 1.29 – 1.49 | 8.92 | **<0.001** |
| Treatment [Flavanols] | 0.97 | 0.07 | 0.85 – 1.12 | -0.37 | 0.710 |
| Treatment [Caffeine] | 1.07 | 0.08 | 0.93 – 1.24 | 0.99 | 0.324 |
| Treatment [Concurrent] | 1.07 | 0.07 | 0.93 – 1.22 | 0.93 | 0.353 |
| **Random Effects** | *Variance* | *Sd* |  |  |  |
| τ_00 Subject_ | 0.50 | 0.71 |  |  |  |
| N _subject_ = 48, Observations = 62197 | | | | | |

Table S3

*GLMM Results for the Effects of Flavanols and Caffeine on T2|T1 Accuracy*

|  | **T2\|T1 Accuracy** | | | | |
| --- | --- | --- | --- | --- | --- |
| **Fixed Effects** | *Odds Ratios* | *std. Error* | *CI* | *z* | *p* |
| Intercept | 3.10 | 0.45 | 2.32 – 4.13 | 7.71 | **<0.001** |
| Lag [Lag 3] | 1.15 | 0.06 | 1.04 – 1.28 | 2.66 | **0.008** |
| Lag [Lag 8] | 2.15 | 0.26 | 1.70 – 2.72 | 6.33 | **<0.001** |
| **Random Effects** | *Variance* | *Sd* |  |  |  |
| τ_00 Subject_ | 1.002 | 1.0008 |  |  |  |
| τ_11 Subject._ _Lag 3_ | 0.09 | 0.30 |  |  |  |
| τ_11 Subject._ _Lag 8_ | 0.65 | 0.81 |  |  |  |
| N _subject_ = 48, Observations = 53962 | | | | | |

Table S4

*LMM Results for the Effects of Flavanols and Caffeine on T2|T1 RT*

|  | **T2\|T1 RT** | | | | |
| --- | --- | --- | --- | --- | --- |
| **Fixed Effects** | *Estimates* | *std. Error* | *CI* | *t* | *p* |
| Intercept | 1034.02 | 31.61 | 972.06 – 1095.98 | 32.71 | **<0.001** |
| Lag [Lag 3] | -97.67 | 7.74 | -112.84 – -82.49 | -12.61 | **<0.001** |
| Lag [Lag 8] | -273.28 | 17.06 | -306.73 – -239.83 | -16.01 | **<0.001** |
| Treatment [Flavanols] | -2.10 | 10.88 | -23.43 – 19.22 | -0.19 | 0.847 |
| Treatment [Caffeine] | -25.26 | 10.86 | -46.54 – -3.98 | -2.33 | **0.020** |
| Treatment [Concurrent] | -24.97 | 10.84 | -46.22 – -3.72 | -2.30 | **0.021** |
| **Random Effects** | *Variance* | *Sd* |  |  |  |
| σ^2^ (Residual) | 41439.43 | 203.57 |  |  |  |
| τ_00 Subject_ | 45501.00 | 213.31 |  |  |  |
| τ_11 Subject._ _Lag 3_ | 2560.49 | 50.60 |  |  |  |
| τ_11 Subject._ _Lag 8_ | 13684.31 | 116.98 |  |  |  |
| N _subject_ = 48, Observations = 44059 | | | | | |

Table S5

*GLMM Results for the Effects of Caffeine and Flavanols on Visual Search Accuracy*

|  | **Visual Search Accuracy** | | | | |
| --- | --- | --- | --- | --- | --- |
| **Fixed Effects** | *Odds Ratios* | *std. Error* | *CI* | *z* | *p* |
| Intercept | 4.87 | 0.46 | 4.05 – 5.87 | 16.67 | **<0.001** |
| Num. of Distractors [20] | 0.62 | 0.02 | 0.59 – 0.66 | -15.99 | **<0.001** |
| Num. of Distractors [26] | 0.42 | 0.01 | 0.40 – 0.45 | -30.14 | **<0.001** |
| **Random Effects** | *Variance* | *Sd* |  |  |  |
| τ_00 Subject_ | 0.40 | 0.63 |  |  |  |
| N _subject_ = 48, Observations = 62208 | | | | | |

Table S6

*LMM Results for the Effects of Caffeine and Flavanols on Visual Search RT*

|  | **Visual Search RT** | | | | |
| --- | --- | --- | --- | --- | --- |
| **Fixed Effects** | *Estimates* | *std. Error* | *CI* | *t* | *p* |
| Intercept | 966.76 | 17.42 | 932.63 – 1000.90 | 55.51 | **<0.001** |
| Num. of Distractors [20] | 56.18 | 2.85 | 50.60 – 61.77 | 19.70 | **<0.001** |
| Num. of Distractors [26] | 104.28 | 2.90 | 98.59 – 109.98 | 35.91 | **<0.001** |
| Treatment [Caffeine] | -16.42 | 7.60 | -31.31 – -1.53 | -2.16 | **0.031** |
| Treatment [Flavanols] | -6.53 | 7.57 | -21.37 – 8.31 | -0.86 | 0.388 |
| Treatment [Concurrent] | -12.34 | 7.66 | -27.36 – 2.68 | -1.61 | 0.107 |
| **Random Effects** | *Variance* | *Sd* |  |  |  |
| σ^2^ (Residual) | 72138.05 | 268.59 |  |  |  |
| τ_00 Subject_ | 13093.00 | 114.42 |  |  |  |
| N _subject_ = 48, Observations = 51630 | | | | | |

Table S7

*LMM Results for the Effects of Caffeine and Flavanols on Visual Working Memory Accuracy*

|  | **Visual Working Memory Accuracy** | | | | |
| --- | --- | --- | --- | --- | --- |
| **Fixed Effects** | *Estimates* | *std. Error* | *CI* | *t* | *p* |
| Intercept | 89.69 | 0.46 | 88.78 – 90.60 | 193.51 | **<0.001** |
| Num. of Items [3] | -19.60 | 0.77 | -21.10 – -18.09 | -25.58 | **<0.001** |
| Num. of Items [2] | -9.33 | 0.56 | -10.42 – -8.23 | -16.74 | **<0.001** |
| **Random Effects** | *Variance* | *Sd* |  |  |  |
| σ^2^ (Residual) | 402.27 | 20.06 |  |  |  |
| τ_00 Subject_ | 8.30 | 2.88 |  |  |  |
| τ_11 Subject._ _Num. of Items [3]_ | 26.17 | 5.12 |  |  |  |
| τ_11 Subject._ _Num. of Items [2]_ | 12.88 | 3.59 |  |  |  |
| N _subject_ = 48, Observations = 57570 | | | | | |
